# Anticancer Effects of Midazolam on Lung and Breast Cancers by Inhibiting Cell Proliferation and Epithelial-Mesenchymal Transition

**DOI:** 10.3390/life11121396

**Published:** 2021-12-13

**Authors:** Hsin-Ling Lu, King-Chuen Wu, Char-Wen Chen, Hong-Kai Weng, Bu-Miin Huang, Ting-Yu Lin, Ming-Hsin Liu, Edmund-Cheung So, Ruey-Mo Lin, Yang-Kao Wang

**Affiliations:** 1 Department of Cell Biology and Anatomy, College of Medicine, National Cheng Kung University, Tainan 70101, Taiwan; angela1827lu@gmail.com (H.-L.L.); bumiin@mail.ncku.edu.tw (B.-M.H.); t96094028@gs.ncku.edu.tw (T.-Y.L.); littlebusters00268@gmail.com (M.-H.L.); 2 Department of Nursing, Chang Gung University of Science and Technology, Chiayi County 61363, Taiwan; kingwutw@cgmh.org.tw; 3 Department of Anesthesiology, Chang Gung Memorial Hospital, Chiayi County 61363, Taiwan; 4 Division of Pulmonary and Critical Care Medicine, Ditmanson Medical Foundation Chia-Yi Christian Hospital, Chiayi County 60002, Taiwan; 04302@cych.org.tw; 5 Institute of Basic Medical Sciences, College of Medicine, National Cheng Kung University, Tainan 70101, Taiwan; s58041056@gs.ncku.edu.tw; 6 Department of Orthopedics, National Cheng Kung University Hospital, College of Medicine, National Cheng Kung University, Tainan 70101, Taiwan; 7 Department of Anesthesiology, An-Nan Hospital, China Medical University, Tainan 70965, Taiwan; 8 Department of Orthopedic Surgery, An-Nan Hospital, China Medical University, Tainan 70965, Taiwan

**Keywords:** midazolam, transforming growth factor β, cancer cell proliferation, epithelium-mesenchyme transition, peripheral benzodiazepine receptor

## Abstract

Despite improvements in cancer treatments resulting in higher survival rates, the proliferation and metastasis of tumors still raise new questions in cancer therapy. Therefore, new drugs and strategies are still needed. Midazolam (MDZ) is a common sedative drug acting through the γ-aminobutyric acid receptor in the central nervous system and also binds to the peripheral benzodiazepine receptor (PBR) in peripheral tissues. Previous studies have shown that MDZ inhibits cancer cell proliferation but increases cancer cell apoptosis through different mechanisms. In this study, we investigated the possible anticancer mechanisms of MDZ on different cancer cell types. MDZ inhibited transforming growth factor β (TGF-β)-induced cancer cell proliferation of both A549 and MCF-7 cells. MDZ also inhibited TGF-β-induced cell migration, invasion, epithelial-mesenchymal-transition, and Smad phosphorylation in both cancer cell lines. Inhibition of PBR by PK11195 rescued the MDZ-inhibited cell proliferation, suggesting that MDZ worked through PBR to inhibit TGF-β pathway. Furthermore, MDZ inhibited proliferation, migration, invasion and levels of mesenchymal proteins in MDA-MD-231 triple-negative breast cancer cells. Together, MDZ inhibits cancer cell proliferation both in epithelial and mesenchymal types and EMT, indicating an important role for MDZ as a candidate to treat lung and breast cancers.

## 1. Introduction

There were more than 19.3 million new cancer cases and 10 million cancer deaths that occurred in 2020 [1]. Although treatment protocols have been developed for various cancers in recent years, there has not been much improvement in cancer mortality rates. The new approach of drug repurposing may present another opportunity to improve outcomes of cancer therapy. There also exist a number of challenges to the treatment of cancer, in particular achieving an understanding of the diverse mechanisms underlying the proliferation and metastasis of cancer cells. The ability of these cells to survive beyond normal life span and to proliferate abnormally has been a major focus of the research into finding appropriate anti-cancer drugs [2]. Previous studies have shown that growth factors such as transforming growth factor-β (TGF-β) possesses tumor-suppressor properties, including cell-cycle arrest and induction of apoptosis in normal epithelial cells [3,4]. In contrast, TGF-β also acts as a tumor promoter in several cancer types due to mutations that taken place during cancer proliferation. This may lead to resistance to the growth inhibitory effects of TGF-β and eventually promote tumorigenesis, increases in metastasis and chemo resistance [5,6].

Tumor metastasis is another difficult task when treating cancer patients, potentially resulting in resistance to chemotherapy, radiation therapy and a high mortality rate [7]. Although metastasis is a complicated process, each step may present an opportunity for the development of new therapeutic approaches. For example, during the early period of metastasis, cancer cells may transform from epithelial cells to mesenchymal cells, known as epithelial-mesenchymal transition (EMT) [8]. Blocking EMT, in addition to administering cancer therapies, may provide more effective treatments. Interestingly, it has been shown that while epithelial cells undergo EMT, they lose their epithelial markers, such as E-cadherin, α-, and β-catenin, and gain mesenchymal markers, including N-cadherin, vimentin, fibronectin, etc. [9,10,11]. In addition to these epithelial surface markers, several transcriptional factors are necessary for EMT, including Zeb1, Snail, Twist, and Slug [11,12,13]. A hallmark of EMT in cancer metastasis is the loss of E-cadherin expression and several important embryogenesis-related genes have been shown to act as E-cadherin repressors [14,15]. Among the different EMT inducers, TGF-β is known to be one of the potent EMT inducers through the activation of mothers against decapentaplegic homolog (Smad) pathway (canonical) or non-Smad signaling (non-canonical) pathways [16]. Therefore, targeting TGF-β-dependent signaling in cancer cell EMT and metastasis shows potential for the development of future therapeutic strategies.

During surgical procedures, anesthesia is required to alleviate patient anxiety and produce analgesia and amnesia. Midazolam (MDZ) is a rapid-onset, short-acting benzodiazepine derivative which has been employed alone or in combinations with other sedative drugs to achieve these purposes [17,18]. It also produces anxiolytic, hypnotic and muscle relaxant effects [19]. The sedative effect of benzodiazepines has been shown to work on the central nervous system by binding to GABA receptors [19]. The peripheral-type benzodiazepine receptor (PBR) has been identified in the kidneys, lungs, ovaries, testes, and adrenal glands, as well as in blood cells, and in glial cells, located primarily on the mitochondrial outer membrane [20,21,22]. Recently, reports have shown that MDZ is a novel therapeutic agent to treat cancer [23]. Administrated in high doses (100 μM and higher), MDZ has been show to induce apoptosis via activation of the mitochondrial pathway in human lymphoma and neuroblastoma cells [24], or regulated endoplasmic reticulum stress, autophagy, and cell cycle to induce apoptosis in Leydig tumor/progenitor cells [25,26]. However, the detail mechanisms by means of which MDZ is involved in the cancer progression and EMT remain largely unknown. Therefore, in this study, we examine how MDZ inhibits both the proliferation of epithelial and mesenchymal cancer cells and the EMT process, as well as the possible molecular mechanisms underlying its inhibitory effects.

## 2. Materials and Methods

### 2.1. Chemicals and Reagents

Midazolam (Dormicum^®^) was purchased from Roche Products Ltd. (Auckland, New Zealand). Transforming growth factor β1 (TGF-β1) was purchased from Peprotech Inc. (Rocky Hill, NJ, USA). RPMI-1640 medium, Dulbecco’s Modified Eagle Medium (DMEM, low glucose), fetal bovine serum (FBS), penicillin/streptomycin, bicinchoninic acid (BCA) protein assay kit, and SuperBlockTM blocking reagent were purchased from Thermo Fisher Scientific (Waltham, MA, USA). 3-(4,5-Dimethylthiazol-2-yl)-2,5-diphenyltetrazolium bromide (MTT), 5-lethyl-2′-deoxyuridine (EdU) cell proliferation kits, Alexa 488-conjugated second antibody, phalloidin-tetramethylrhodamine (TRITC), horseradish peroxidase conjugated secondary antibody and 4’, 6-diamidino-2-phenylindole (DAPI) were purchased from Abcam (Cambridge, England). Transwell culture inserts were purchased from ibidi (Gräfelfing, Germany). Matrigel^®^ growth factor reduced basement membrane matrix was purchased from Corning (Corning, NY, USA). Anti-integrin β1, anti-vinculin antibodies, a radioimmunoprecipitation assay buffer (RIPA buffer, 10×) was purchased from Merck KGaA (Darmstadt, Germany). Protease and phosphatase inhibitor cocktails were purchased from BioTool (Keperra QLD, Australia). Anti-fibronectin antibody was purchased from BD Biosciences (San Jose, CA, USA). Anti-vimentin was purchased from Agilent (Santa Clara, CA, USA). Anti-phospho-Smad2/3; anti-Smad3 antibodies were purchased from Cell Signaling (Danvers, MA, USA). Enhances Chemiluminescence (ECL) substrate was purchased from Bio-Rad (Hercules, CA, USA). All other chemicals were purchased from Merck KGaA, unless otherwise specified.

### 2.2. Cell Culture and Treatment

The A549 human lung alveolar adenocarcinoma cell line, the human breast cancer cell line MCF-7 (A549 cell, cat# 60074; MCF-7 cat# 60436) and the human normal mammary epithelial cell line H184B5F5/M10 (M10 cell, cat# 60197) were purchased from the Bioresource Collection and Research Center (BCRC, Hsinchu, Taiwan). The A549 cells were cultured in RPMI-1640 medium supplemented with 10% FBS, 100 U/mL of penicillin, and 100 μg/mL of streptomycin. The MCF-7 breast cancer cells were cultured in DMEM (low glucose) supplemented with 10% FBS, 10 μg/mL insulin, 100 U/mL of penicillin, and 100 μg/mL of streptomycin. The MDA-MB-231 human breast cancer cell line was purchased from American Type Culture Collection (CRM-HTB-26; Manassas, VA, USA) and was maintained in RPMI-1640 medium supplemented with 10% FBS, 100 U/mL of penicillin, and 100 μg/mL of streptomycin. Human bone marrow derived mesenchymal stem cells were purchased from Lonza (Walkersville, MD, USA) and was maintained in DMEM (low glucose) supplemented with 10% FBS (with selected lot number), 100 U/mL of penicillin, and 100 μg/mL of streptomycin. All the cell lines were maintained at 37 °C in a humidified CO2 (5%) incubator. In the experiments described in the following sections, the A549 and MCF-7 cells were serum starved (0.5% FBS) for overnight and then treated with TGF-β1 (10 ng/mL) and different concentrations of MDZ (0, 5, 10, and 20 μM). In addition, MDA-MB-231 cells were also serum starved (0.5% FBS) for overnight and then treated with different concentrations of MDZ (0, 5, 10, and 20 μM).

### 2.3. MTT Assay

Cells (2 × 10^3^ cells/well) were seeded overnight in a 96-well plate at 2 × 10^3^ cells/well. After treatment, the cells were fed with MTT solution (Stock: 5 mg/mL, 20 µL/well) on the designated day and incubated for 3 h. The solution was then aspirated, and 100 µL of dimethyl sulfoxide was added to each well, mixed and incubated at 37 °C for another 30 min. The absorbance of each sample was then measured at 570 nm with a microplate reader (μQuant Universal Microplate Reader, Bio-Tek, Winooski, VT, USA).

### 2.4. EdU Cell Proliferation Assay

Cells were seeded overnight in 35 mm dishes or 6-well plates at 8 × 10^4^ cells/well. EdU solution (10 μM) was added to the culture medium and incubated. The medium was then removed, and the treatments were applied as indicated in each sample. At the designated times, the cells were washed and then fixed with 75% ethanol, followed by incubation with assay cocktail. The immunofluorescence images were taken with an Olympus immunofluorescence microscope (IX-71, inverted, Olympus, Tokyo, Japan).

### 2.5. Wound Healing Assay

The A549 and MCF-7 cells were seeded in 6-well plates at 3–5 × 10^5^ cells/well until confluence was reached. A scratch was made with a 200 μL micropipette tip. In addition, the MDA-MB-231 cells were seeded in cell culture insert to create a 500 μm-wide wound. The cells were then treated with designated medium and incubated for 24 h. Images of the wounds were captured immediately after adding of the medium and 24 h after doing so with a phase contrast microscope (Nikon, TS-100). The images were analyzed with ImageJ software (National Institute Health, Bethesda, MD, USA).

### 2.6. Transwell Migration and Invasion Assay

Transwell cell migration was performed using Transwell chamber inserts with membranes with a pore size of 8 μm pores in 24-well plates (Corning, Corning, NY, USA). Cells were seeded in a 35 mm dish, and then, in some cases, they were treated overnight with TGF-β (10 ng/mL) (TGF10) with the addition of MDZ (20 μM, MDZ, T + MDZ). In other cases, the cells were treated with TGF-β without MDZ, and finally neither TGF-β nor MDZ were applied in certain cases. The treated cells were re-suspended in 200 μL serum-free medium and were seeded onto the chamber insert at 1 × 10^4^ cells/chamber. The lower chamber was filled with medium containing chemo-attractants. After 12 h, the upper chamber was removed from the 24-well plate, and cells on the membrane were fixed with 4% paraformaldehyde for 15 min, and then stained with 0.5% Crystal Violet. All cells on the upper surface of the membrane were removed with a cotton swab, and the cells on the other side were visualized with a light microscope. Cell invasion was performed using a Transwell chamber, coated with 60 μL of Matrigel (300 μg/mL) and then incubated overnight. After treatment, cells were suspended in 200 μL of serum-free medium and seeded onto the upper chamber at 1 × 10^5^/chamber. The lower chamber was filled with medium containing chemo-attractants. At the designated time, the upper chambers were removed from the 24-well plate, and the cells on the membrane were fixed with 4% paraformaldehyde for 15 min, and then stained with 0.5% Crystal Violet for 10 min. All cells on the upper surface of the membrane were removed by a cotton swab, and cells on the other side were visualized with a light microscope. Three fields of each treatment were randomly selected for cell count.

### 2.7. Western Blotting

Cells were harvested to prepare a cell lysate using a RIPA lysis buffer (25 mM Tris, pH 7.4, 150 mM NaCl, 0.1% SDS, 0.5% sodium deoxycholate, 1% Triton X-100) with protease and phosphatase inhibitor cocktails. The cell lysate was centrifuged and the supernatant was collected and stored at −80 °C. The protein concentration was measured by BCA protein assay performed according to the manufacturer’s instructions. The sodium dodecyl sulfate–polyacrylamide gel electrophoresis system was used to separate 10–20 μg of protein in each sample, which was then transferred using electroblotting onto a polyvinylidene difluoride (PVDF) membrane. The PVDF membrane was incubated with SuperBlock^TM^ or 5% bovine serum albumin (for the detection of phospho-protein) in Tris-buffered saline with 0.1% Tween-20 at room temperature for 1 h. The membrane was then incubated overnight with a primary antibody, such as anti-fibronectin, anti-integrin β1, anti-vinculin, anti-vimentin, anti-phospho-Smad2/3, or anti-Smad3, at 4 °C, followed by incubating with HRP conjugated secondary antibodies. After incubation, the final immunocomplex was visualized using fluorography (FluroChem R, San Jose, CA, USA) with ECL substrate. The fluoroscopy images were then captured using the BioSpectrum Imaging System (UVP, BioLiteTM MultiSpectral Light Source and VisionWorks^®^ LS software. (The Core Research Laboratory, College of Medicine, National Cheng Kung University).

### 2.8. Immunofluorescence Staining

The MCF-7 and A549 (1.5 × 10^4^) cells were seeded on fibronectin (20 μg/mL)-coated 22 × 22 mm coverslips. After treatment, the samples were fixed by 4% paraformaldehyde for 10 min, permeabilized with 0.1% Triton X-100 for 10 min and then soaked in 3% BSA/1X phosphate-buffered saline (PBS) for 1 h, after which they were incubating overnight with a primary antibody at 4 °C. Samples were rinsed with PBS twice and incubated with Alexa 488-conjugated secondary antibody and phalloidin-TRITC, and then images were taken with an Olympus epifluorescence microscope or a confocal microscope (FV-1000, Olympus). The cell nuclei were counterstained with DAPI.

### 2.9. Statistical Analyses

All quantified results from three to five independent experiments are expressed as mean ± standard deviation of three to five independent experiments. Statistical analyses were performed with one-way ANOVA followed by Tukey’s honestly significant difference (HSD) test Prism software (GraphPad, San Diego, CA, USA). Statistical significance was set at *p* values < 0.05.

## 3. Results

### 3.1. Midazolam Inhibits TGF-β-Induced Cancer Cell Proliferation

**Figure 1:**
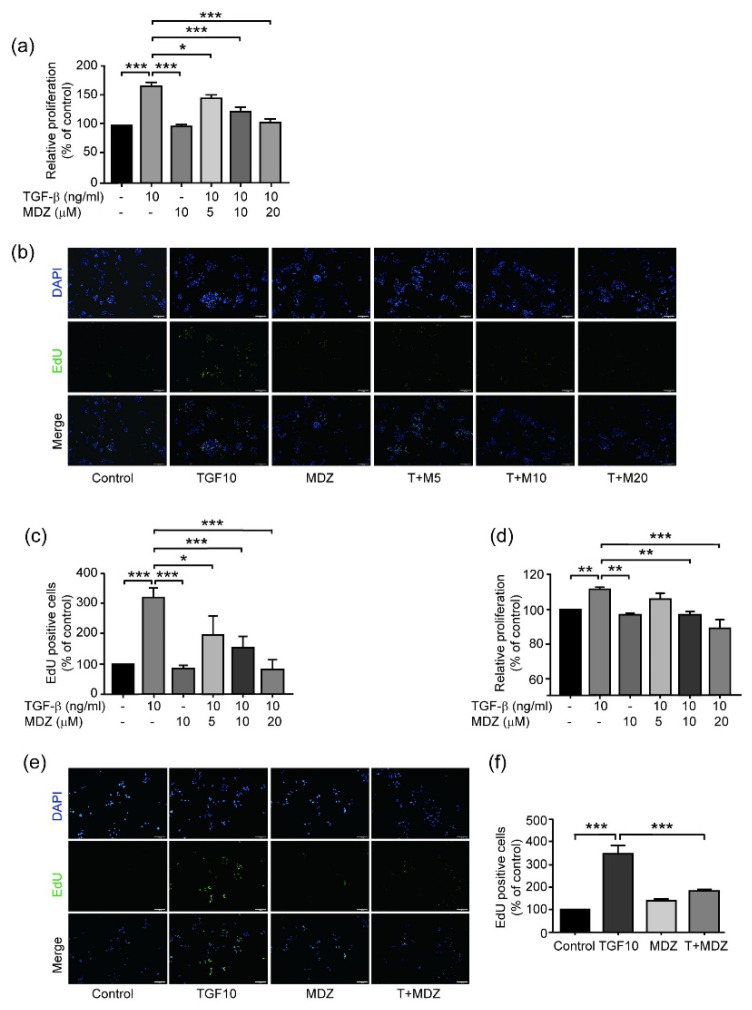
Midazolam inhibits TGF-β1-induced cancer cell proliferation. A549 cells (6 × 10^3^ cells/cm^2^) were treated without (Control) or with TGF-β1 (10 ng/mL) (TGF-β) in the presence of various concentrations of midazolam (5, 10 and 20 μM) for 48 h. Treatment of MDZ alone at the concentration of 10 μM (MDZ10) was used as a negative control. (**a**) MTT assay results for A549 cells. (**b**) Representative images of EdU incorporation of A549 cells. (**c**) Quantitative results of (**b**). (**d**) MCF-7 cells (8 × 10^3^ cells/cm^2^) were treated without (Control) or with TGF-β1 10 ng/mL (TGF-β) in the presence various concentrations of MDZ (5, 10 and 20 μM) for 48 h. Cell viability was accessed by MTT assay. (**e**) Representative images of EdU incorporation of MCF-7 cells. MCF-7 (8 × 10^3^ cells/cm^2^) were treated without (Control) or with TGF-β1 10 ng/mL (TGF10) and co-treated with MDZ (20 μM). Treatment of MDZ alone at the concentration of 10 μM (MDZ10) was used as a negative control. (**f**) Quantitative results of (**e**). Results of three independent experiments are expressed as means ± standard deviation. *: *p* < 0.05; **: *p* < 0.01; ***: *p* < 0.001. Scale bar: 100 μm.

To elucidate the effects of MDZ on cancer cell behaviors, we first investigated the proliferation of different carcinoma cell lines. The A549 human lung cancer cell line and the MCF-7 breast cancer cell line (luminal A, estrogen receptor positive) were seeded at 6 × 10^3^ cells/cm^2^, serum starved and treated with TGF-β (10 ng/mL) and with or without different doses of MDZ (5, 10, and 20 μM). After 48-h treatment, the proliferation was examined using MTT assays. The results showed that TGF-β significantly increased A549 cells proliferation compared to the control, whereas treatment with MDZ (10 μM) alone showed no effect on A549 cell proliferation. In fact, MDZ inhibited the TGF-β-induced proliferation of A549 cell in a dose-dependently manner (Figure 1a). To further confirm the inhibitory effect of MDZ on cell proliferation, we performed an EdU proliferation assay. Treatment with TGF-β (10 ng/mL) significantly induced the incorporation of EdU in A549 cells compared to the untreated control (Figure 1b,c). Treatment of MDZ alone showed no effect on incorporation of EdU in cells, whereas co-treatment of MDZ dose dependently inhibited TGF-β-induced incorporation of EdU. Similar results were found with MCF-7 cells (Figure 1d–f). These results suggest that MDZ inhibits the TGF-β-induced proliferation of carcinoma cells.

Since the administration of anticancer drugs is allowed to inhibit proliferation or increase apoptosis of cancer cells at concentrations that do not significantly affect non-tumoral cells, we then tested whether treatment of MDZ on normal cell lines resulted in a cytotoxic effect. We performed two normal cell lines, one was the primary human bone marrow-derived mesenchymal stem cells (hMSC) and the other one was the normal human mammary epithelial cells (M10). These cells were seeded overnight and then treated with MDZ with different concentrations (5, 10, 20, 50, and 100 M) for 48 h and then the cell viability was examined using MTT assay. In hMSCs, treatment with MDZ at doses below 50 M did not affect cell viability, whereas MDZ at a dose of 100 M significantly decreased cell viability. In M10 cells, treatment with MDZ at both 50 and 100 M significantly decreased cell viability (Supplement Figure S1). This result suggested that treatment with MDZ at the does of 20 M did not result in cytotoxic effects in normal cells.

### 3.2. Midazolam Inhibits TGF-β-Induced Cancer Cell Migration

**Figure 2:**
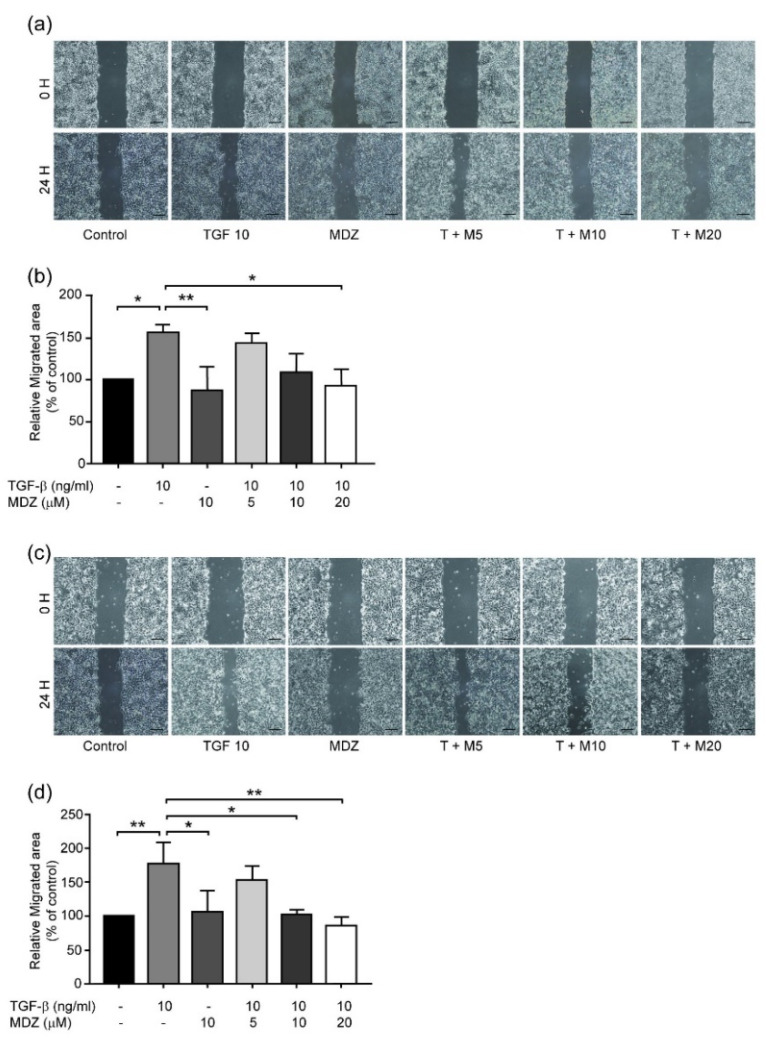
Midazolam inhibits TGF-β1-induced cancer cell migration. A549 and MCF-7 cells were seeded and grown to sub-confluence and then treated without (Control) or with TGF-β1 (10 ng/mL) (TGF 10) in the presence of various concentrations of MDZ (5, 10 and 20 μM, denoted as T + M5, T + M10 and T + M20, respectively) after creating a wide wound. Treatment with MDZ alone at a concentration of 10 μM was used as a negative control. (**a**,**c**) Representative images of wound areas of (**a**) A549 and (**c**) MCF-7, respectively, were captured at the designated time (0 H, 24 H). (**b**,**d**) Quantitative results of wound areas of (**a**,**c**), respectively, were calculated and the results were normalized with the untreated control. The results of three independent experiments are presented as means ± standard deviation. *: *p* < 0.05; **: *p* < 0.01. Scale bar: 100 μm.

**Figure 3:**
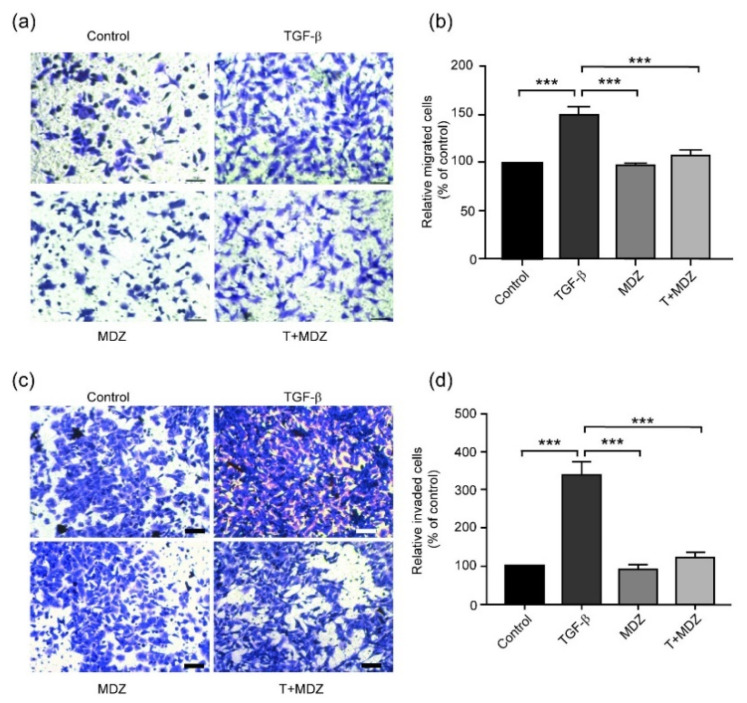
Midazolam inhibits TGF-β1-induced Transwell migration and invasion. A549 cells were pre-treated overnight without (Control) or with TGF-β1 (10 ng/mL) (TGF-β) in the absence or presence of MDZ (20 μM) (denoted as MDZ and T + MDZ, respectively) and seeded at the density of 1 × 10^4^ cells/upper chamber. The lower chamber contained a chemoattractant. (**a**) After 12 h, cells on the lower surface of the Transwell inserts were fixed and stained and the representative images were captured. (**b**) Quantitative results of (**a**). MCF-7 cells were pre-treated overnight without (Control) or with TGF-β1 10 ng/mL (TGF-β) in the absence or presence of MDZ (20 μM) (denoted as MDZ and T + MDZ, respectively) and seeded at density of 1 × 10^5^ cells onto Matrigel-coated upper chamber. (**c**) After 24 h, cells on the lower surface of the Transwell inserts were fixed and stained and the images were captured. (**d**) Quantitative results of (**c**). Results of three independent experiments are presented as means ± standard deviation. ***: *p* < 0.001. Scale bar: 50 μm.

TGF-β can not only increase the proliferation of cancer cells but also induce their migration and invasion [16]. Therefore, we next sought to elucidate whether MDZ inhibits the TGF-β-induced migration and invasion of cancer cells. We first used a wound healing assay to analyze the migration capacity of the A549 and MCF-7 cells after treatment with TGF-β. The A549 cells were seeded and let the cell grow up to sub-confluence. After the cells were serum starved overnight, a wide wound was created and the cells were treated with TGF-β (10 ng/mL) and without or with different doses of MDZ (5, 10, and 20 μM). After 24-h treatment, there was no significant wound closure in control cells. Treatment with TGF-β-accelerated wound closure; however, this TGF-β-induced cell migration was inhibited in a dose dependent manner when the cells were co-treated with MDZ (Figure 2a,b). Interestingly, treatment with MZD alone (10 μM) did not alter basal cell migration (Figure 2a,b). Similar results were found in MCF-7 cell (Figure 2c,d), showing that MDZ inhibited TGF-β-induced cell migration in a dose dependent manner. Given that cancer cell migration happens in 3D and a single cell can migrate directionally in response to chemoattractant stimulation [27], we performed a Transwell migration assay. The A549 cells were seeded in a 35 mm dish, serum starved, treated overnight with or without MDZ, and then re-suspended and seeded onto the chamber insert. The lower chamber was filled with medium containing TGF-β (10 ng/mL). The migrated cells were then stained and counted. The results showed that TGF-β induced a significant increase of cell migration in Transwell assay which was inhibited by treatment with MDZ (Figure 3a,b). These results suggest that TGF-β-induced migration of cancer cells can be inhibited by MDZ.

### 3.3. Midazolam Inhibits TGF-β-Induced Cell Invasion

To further investigate whether MDZ inhibits cancer cell invasion, we performed a Transwell assay with a layer of Matrigel coating on the membrane. The A549 cells were seeded on the upper chamber at 1 × 10^5^ cells/well and tested to determine whether MDZ inhibited the TGF-β-induced invasion of A549 cells. The results showed that in control cells, a certain amount of A549 cells invaded from the upper chamber to the lower chamber. Treatment with TGF-β (10 ng/mL) increased A549 cell invasion whereas treatment with MDZ alone did not affect the basal invasion capability of the A549 cells. This TGF-β-induced cell invasion was significantly reduced by co-treatment of MDZ (20 μM) (Figure 3c,d). These results indicated that MDZ inhibits TGF-β-dependent cell migration and cell invasion.

### 3.4. Midazolam Suppresses TGF-β-Induced EMT in Cancer Cells

**Figure 4:**
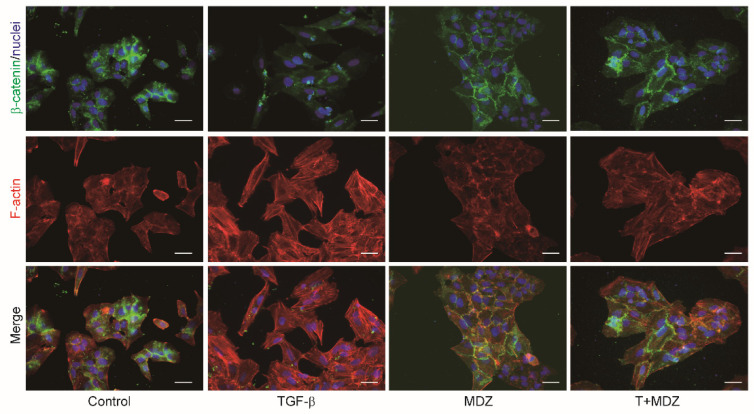
Midazolam inhibits TGF-β-induced epithelial-mesenchymal transition in A549 cells. Representative images of A549 cells were grown on 20 μg/mL fibronectin-coated 22 × 22 mm coverslips and treated without (Control) or with TGF-β1 (10 ng/ml) (TGF-β) in the absence or presence of MDZ (20 μM) (denoted as MDZ and T + MDZ, respectively) for 24 h. The localization of β-catenin and F-actin were determined by immunofluorescence. The β-catenin was stained with Alexa-488 (green) and F-actin was stained with phalloidin-TRITC (red). Nuclei were stained with DAPI (blue). Scale bar: 20 μm.

The above results presented in the preceding sections show that MDZ has an inhibitory effect of MDZ on TGF-β-dependent proliferation, migration and invasion of cancer cells. It has been suggested that TGF-β is a potent inducer of EMT in cancer [28], and our results showed that MDZ inhibited TGF-β-induced migration and invasion of both A549 and MCF-7 cells. However, whether MDZ inhibits the epithelial markers of both cell types and promotes EMT remains unclear. Consequently, the next step in this study was to analyze the localization of the epithelial marker β-catenin by using immunofluorescence after treatment with TGF-β. The A549 and MCF-7 cells (1.5–2 × 10^4^ cells) were allowed to attach and spread on fibronectin-coated coverslips, after which they were serum starved overnight. The cells were then treated with TGF-β (10 ng/mL) both with and without the addition of MDZ (20 μM). After 24 h, the cells were fixed to inspect the epithelial marker β-catenin and the organization of F-actin. The results showed that in control cells, β-catenin was observed at cell–cell junctions and co-localized with F-actin in A549 (Figure 4). Treatment with TGF-β inhibited the localization of β-catenin at cell–cell junctions and caused a reorganization of actin filaments from cell–cell junctions to thick contractile stress fibers, which were then distributed to the bottom of the cells (Figure 4). This result suggested that the cells lost their epithelial phenotype as they separated from each other, which is known as the EMT process. Treatment with MDZ alone did not affect the localization of β-catenin or the organization of F-actin. Treatment with both TGF-β and MDZ, on the one hand, diminished the effect of TGF-β-induced EMT, and restored the localization of β-catenin to the cell–cell adhesion sites, and decreased thick contractile stress fibers (Figure 4). Similar results were also found in MCF-7 cells, where MDZ returned β-catenin localization back to the cell–cell adhesion sites in the presence of TGF-β, whereas the organizations of F-actin did not change significantly (Supplemental Figure S2). Together, these results indicated that MDZ inhibits TGF-β-induced EMT in both A549 and MCF-7 cells.

### 3.5. Midazolam Inhibits TGF-β-Induced Smad Signaling

**Figure 5:**
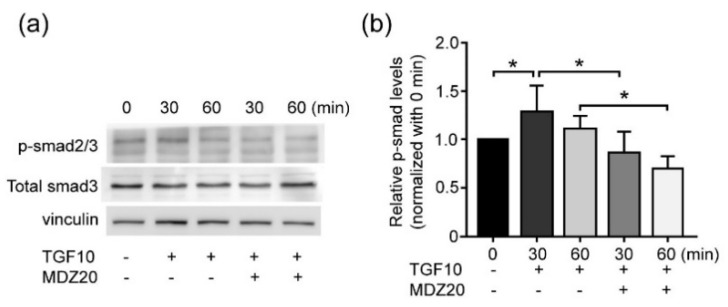
Midazolam inhibits TGF-β-induced Smad phosphorylation. The MCF-7 cells were seeded at 2 × 10^4^ cells/cm^2^ and then treated with TGF-β1 (10 ng/mL) (TGF10) without or with the presence of MDZ (20 μM) (MDZ20). The cells were harvested to prepare the cell lysate at the designated times. (**a**) The levels of total Smad3 and phosphor-Smad2/3 (p-smad2/3) were determined by Western blotting and the protein levels of vinculin was used as a loading control. (**b**) Quantification results of (**a**). The results of three independent experiments are expressed as means ± standard deviation. *: *p* < 0.05.

The phosphorylation of Smads is one of the major signaling pathways in TGF-β-induced EMT [29]. To further explore the specific mechanisms underlying the inhibitory effects of MDZ on cancer cells, we examined whether MDZ affects the TGF-β-induced phosphorylation of Smads. The MCF-7 cells were serum starved, and then stimulated with TGF-β (10 ng/mL), with the addition of MDZ (20 μM). After being treated for 30 min and 60 min, cells were harvested to prepare the cell lysate. The total phosphorylated levels and of Smad2/3 were determined by Western blotting. The results showed that the levels of phospho-Smad2/3 was upregulated by TGF-β after 30 min, and the signal decreased after 60 min. This TGF-β-induced Smad phosphorylation was inhibited by co-treatment with MDZ (Figure 5a,b). This result suggests that the inhibitory effects of MDZ on the TGF-β-induced proliferation and EMT in cancer cells acts through the suppression of the Smad signaling pathway.

### 3.6. Midazolam Inhibits Cancer Cell Proliferation via Peripheral Benzodiazepine Receptor

**Figure 6:**
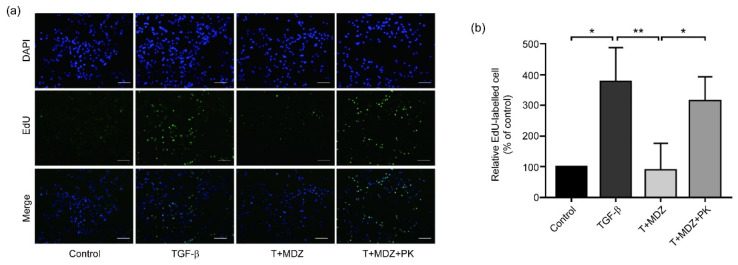
Peripheral benzodiazepine receptor antagonist PK11195 reverses Midazolam-inhibited cancer cell proliferation. The A549 cells were seeded and pre-treated without or with PK11195 (30 μM) for 1 h. After removing the medium, the A549 cells were treated with TGF-β1 (10 ng/mL) (TGF-β) alone, or with MDZ (20 μM) (denoted as T + MDZ) and PK11195 (30 μM, denoted as T + MDZ + PK) for 48 h. (**a**) Representative images of EdU proliferation assay. (**b**) The quantitative results of EdU proliferation assay. Results of three independent experiments are expressed as means ± standard deviation. *: *p* < 0.05; and **: *p* < 0.01. Scale bar: 100 μm.

To elucidate the molecular mechanisms underlying the inhibitory effect of MDZ on cancer cells, we examined the role of the PBR, which has been found to mediate the effects of benzodiazepines in peripheral tissues [21,30]. To determine whether MDZ exerted its inhibitory effects through the intermediary of the PBR, the PBR antagonist, PK11195 was used [31,32,33]. The A549 cells were seeded and pre-treated with PK11195 (30 μM) for 1 h after being serum starved. The cells were then treated with TGF-β (10 ng/mL), MDZ (20 μM) and PK11195 (30 μM) for another 48 h. We analyzed proliferation of the A549 cells using an EdU proliferation assay. As shown in Figure 6, the inhibitory effect of MDZ on TGF-β-induced proliferation was reversed by the treatment with PK11195 (Figure 6a,b). These results indicates that the inhibitory effect of MDZ on TGF-β-induced proliferation of A549 cells may be mediated by the PBR.

### 3.7. Midazolam Inhibits Proliferation and Mesenchymal Marker Expression of MDA-MB 231

**Figure 7:**
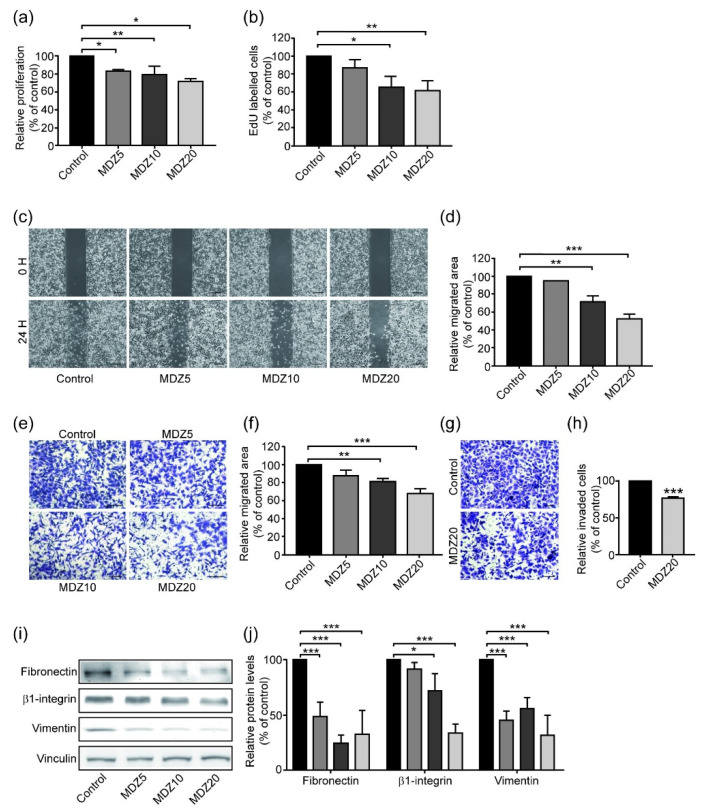
Midazolam inhibits proliferation, migration, invasion and protein levels of mesenchymal markers in MDA-MB-231 cells. MDA-MB-231 cells were seeded at 6 × 10^3^ cells/cm^2^ and treated without (Control) or with MDZ (5, 10 and 20 μM, denoted as M5, M10 and M20, respectively) for 48 h. The cell proliferation was evaluated using (**a**) MTT assay and (**b**) EdU proliferation assay. Results of three independent experiments are expressed as means ± standard deviation. *: *p* < 0.05; **: *p* < 0.01. MDA-MB-231 cells were seeded and grown to sub-confluence and a wound was created using an insert. Cells were then treated without (Control) or with MDZ (5, 10 and 20 μM, denoted as M5, M10 and M20, respectively) for 12 h. (**c**) Images of the wound areas were captured at the designated time. (**d**) The quantitative results of wound healing assay of (**c**). The results were normalized with the untreated control. Results of three independent experiments are expressed as means ± standard deviation. **, *p* < 0.01; ***, *p* < 0.001. Scale bar: 100 μm. MDA-MB-231 cells were pretreated overnight with various doses of MDZ (5, 10 and 20 μM, denoted as M5, M10 and M20, respectively) and then seeded at 1 × 10^4^ cells/chamber in the upper chamber. The lower chamber contained a chemoattractant. (**e**) After 12 h, cells on the lower surface of the chamber were fixed and stained, and the images were taken. (**f**) The quantitative result of (**e**) was calculated and the results were normalized to untreated control. Results of three independent experiments are expressed as means ± standard deviation. **: *p* < 0.01; ***: *p* < 0.001. Scale bar: 50 μm. MDA-MB-231 cells were pre-treated overnight without (control) or with MDZ (20 μM, denoted as M20) and then seeded at 1 × 10^5^ cells/chamber in a Matrigel-coated upper chamber. (**g**) After 24 h, cells on the lower surface of chamber were fixed and stained, and the images were taken. (**h**) The quantitative result of (**g**) was calculated and the results were normalized to untreated control. Results of three independent experiments are expressed as means ± standard deviation. ***: *p* < 0.001 vs. control. Scale bar: 50 μm. (**i**) MDA-MB-231 were seeded at 2 × 10^4^ cells/cm^2^, treated with various doses of MDZ (5, 10, and 20 μM) for 24 h. Western blotting was performed to analyze the levels of mesenchymal markers, such as fibronectin, β1-integrin and vimentin. The protein levels of vinculin were used as a loading control. (**j**) Quantitative results of (**i**). Results of three to five independent experiments are expressed as means ± standard deviation. *: *p* < 0.05; and ***: *p* < 0.001.

Given the inhibitory effects of MDZ on the TGF-β-induced proliferation and EMT in A549 and MCF-7 cells, we were interested to elucidate whether MDZ also possessed such an inhibitory effects on metastatic cell line, such as MDA-MB-231 cells, which is known to be a triple-negative, basal-B type breast cancer cell [34,35]. Indeed, MDA-MB-231 cells are known to be an aggressive breast cancer cell line which shows a typical EMT phenotype [36]. To identify any connection between MDZ and MDA-MB-231 cells, we tested whether MDZ affected the proliferation and malignancy of MDA-MB-231 cells. The results of MTT and EdU proliferation assays showed that MDZ inhibited MDA-MB-231 cell proliferation in a dose dependent manner (Figure 7a,b). By using wound healing and Transwell migration assays, we found that MDZ inhibited MDA-MB-231 cell migration in a dose dependent manner (Figure 7c–f). In addition, treatment with MDZ inhibited MDA-MB-231 cell invasion (Figure 7g,h). These results indicated that in an aggressive cell line that has already gone through EMT process, MDZ inhibits the proliferation, migration and invasion of cancer cells. Therefore, we then analyzed the mesenchymal markers of the MDA-MB 231 by Western blotting. MDA-MB 231 cells were seeded at 2 × 10^4^ cells/cm^2^ and treated with different dosages of MDZ (5, 10, and 20 μM). After 24 h, the cell lysates were collected. The results showed that the levels of such mesenchymal markers, such as fibronectin, vimentin and β1-integrin, in the MDA-MB 231 cells decreased after treatment with MDZ (Figure 7i,j). Overall, the results showed that MDZ inhibits TGF-β-induced proliferation and EMT of A549 and MCF-7 cancer cells. In an EMT cancer cell type, such as MDA-MB 231 cells, treatment of MDZ alone inhibits proliferation, migration, invasion and EMT process.

## 4. Discussion

In this study, we investigated the inhibitory effects of MDZ on cancer proliferation and EMT. It has been reported that benzodiazepine derivatives, such as clonazepam and diazepam, are carcinogens in previous studies [37,38], one of which [38], a longitudinal population-based case-control study, showed that these benzodiazepine derivatives significantly increased the incidence of brain, colorectal and lung cancers and promoted the metastasis of breast cancer cells [37,38]. These cohort studies only provided data on correlations between the application of these benzodiazepine derivatives and the incidence of cancer progression. Our study, provided direct evidence to show that MDZ inhibits proliferation, migration, invasion and EMT in TGF-β responsive cancer cell lines A549 and MCF-7. This MDZ inhibitory effect was also found in an aggressive EMT MDA-MB-231 cell line. Most importantly, the inhibition by MDZ of cancer proliferation was mediated by the PBR, as proven by the fact that the PBR antagonist (PK11195) reversed the inhibitory effects on cell proliferation. This PBR-mediated inhibitory effect of MDZ could also be observed with MDA-MB-231 cells (data not shown). These results indicated that the PBR is necessary for MDZ to exert its inhibitory effects on TGF-β-induced cancer cell behaviors. Previous studies have demonstrated that specific PBR ligand-induced apoptosis and cell cycle arrest in cancer cells induced chemosensitization in these cells when given a combination of treatments [39,40]. In addition, numerous studies have indicated that PBR was involved in breast, brain, and prostate tumor progression [41,42,43]. These studies supported our findings that MDZ inhibits TGF-β-induced proliferation and migration of cancer cells. Hardwick et al. [42] demonstrated that the extent of PBR-ligand binding and the levels of PBR in MDA-MB 231 were higher than both MCF-7 and normal breast tissue. Furthermore, PBR in MDA-MB 231 cells have been shown to localize primarily in and around the nucleus, whereas this occurred in the cytoplasm of the MCF-7 cells and normal breast tissue [42,44]. The different localizations of PBR in different cell lines may be the result of different responses to the PBR ligand. In MCF7 cells, our findings indicated that the proliferation and migration of cells were regulated by TGF-β and the phosphorylation of its downstream signaling molecule Smad2/3. This inhibition by MDZ of the Smad phosphorylation induced by TGF-β was mediated by PBR, a process that has also been observed in mesenchymal stem cells [33]. The cytosolic localization of PBR may bind with its ligand directly and so further influences the downstream signaling events that happen in the cytosol. This evidence may explain the different inhibitory effect of MDZ in MDA-MB 231 and MCF-7 cells.

Although MDZ is safe when used during surgery, in high doses, it has been shown to potentially lead to cytotoxicity in normal cells and even endanger the life of our patients [17,45]. In clinical usage, the dose for sedation in adult patients without other adjuvants may be as high as 0.6 mg/kg. In extreme conditions, like the treatment of refractory status epilepticus, the maximum dosage of MDZ may reach 0.2 mg/kg/h or 244 mg/day by intravenous infusion [46]. However, treatment with such extremely high dosages may cause respiratory depression or cardiac arrest, and usually requires ventilation support and intensive care for patients [47]. In addition, in human normal oral epithelial cells, treatment with MDZ has been shown to exhibit dose-dependent cytotoxicity with concentrations higher than 60 μM and to cause around 50% cell death [45]. In our experience, normal cells start undergoing death or cell lysis (in vitro) when dosage of MDZ is above 50 μM (Supplemental Figure S1). Therefore, to reduce off-target effects, we chose lower doses of MDZ. Our results suggested that MDZ may benefit the treatment of cancer by its potential inhibitory properties about cell proliferation, migration and EMT without affecting cell viability. However, enhancing the efficacy of this use of MDZ without increasing its dose may be our next challenge. We speculate that combination therapy, a treatment that combines two or more therapeutic agents, may be the answer to this challenge. This approach was first used in 1965, when researchers combined two chemotherapy agents to treat leukemia [48]. Other studies applying this approach have shown that certain combinations have a higher efficacy to treat cancer cells and may decrease the toxicity to the body [49,50,51]. Cisplatin, etoposide and taxol are well-known chemotherapeutic agents that are widely applied to the treatment of lung cancer and pancreatic cancer [49,52]; however, severe side effects may accompany the administration of high doses of these agents, which also presents potential challenges in clinical therapy. To address all these challenges, designing a new cancer treatment protocol that includes MDZ may provide an alternative way to offer more efficient treatments for cancer patients while causing no harm to our normal tissues.

It has been shown that TGF-β is involved in both normal mammalian cell development, abnormal cancer progression, and the EMT process [53,54]. Our results showed that MDZ inhibited TGF-β-induced smad2/3 phosphorylation in MCF-7 cells, which is associated with the induction effect of cancer signaling pathways. In addition to acting through canonical Smad-dependent pathway, TGF-β has also been shown to act through non-canonical Smad-independent signaling pathways [55]. TGF-β activates the Jun N-terminal kinases (JNKs) and p38 mitogen-activated protein kinases (p38) signaling pathways as a result of the TGF-β receptor causing TNF Receptor Associated Factor 6 (TRAF6) to activate transforming growth factor beta-activated kinase 1 (TAK1). In addition, upon binding of TGF-β to its receptor TβRI, it subsequently recruits and phosphorylates Shc, which in turn activates the ERK/MAP kinase signaling pathways [56]. Regarding EMT in cancer cells, TGF-β can activate the ERK1/2 and p38 MAPK signaling pathway, causing the activation of another EMT-related transcription factor Snail, which in turn mediates EMT-related transcription. Therefore, this TGF-β-mediated, non-canonical pathway has become another potential target for the development of cancer therapies [57,58]. Our results showed that MDZ inhibited the proliferation, migration, invasion and expression of mesenchymal markers of MDA-MB 231. The mesenchymal phenotype of these cancer cells implies that their proliferation and their abilities to migrate and invade are not only induced by TGF-β. Given that the malignancy of MDA-MB 231 cells is not limited to the activity of a distinct pathway, this result provided us the idea that the MDZ inhibitory effect in cancer cells not only work through canonical Smad-dependent pathway, but also involve multiple signal pathways that are related to cancer proliferation and the EMT process.

## 5. Conclusions

In summary, we have provided a potential therapeutic strategy using MDZ with both epithelial and mesenchymal types of cancer cells, and we have proven that MDZ can inhibit the TGF-β-mediated cell proliferation and EMT process in cancer. This MDZ inhibition of cancer cell behavior may act through the PBR. This study may provide a novel therapeutic strategy that repositions an existing drug to be used in the treatment of cancer cell proliferation and EMT.

## Data Availability

The datasets used and/or analyzed during the current study are available from the corresponding author on reasonable request.

## Supplementary Materials

The following are available online at https://www.mdpi.com/article/10.3390/life11121396/s1, Figure S1: High dose treatment of MDZ inhibits proliferation of normal cells, Figure S2: Midazolam inhibits TGF-β-induced epithelial-mesenchymal transition in MCF-7 cells.
