# Supplementary material for: Anticancer Effects of Midazolam on Lung and Breast Cancers by Inhibiting Cell Proliferation and Epithelial-Mesenchymal Transition
Source: Life (Basel). 2021 Dec 13;11(12):1396. doi: 10.3390/life11121396 (PMC8703822; doi:10.3390/life11121396)
Supplement: Supplementary file 1 [file life-11-01396-s001.zip › life-1462610-supplementary.pdf]

Supplemental Figure S1

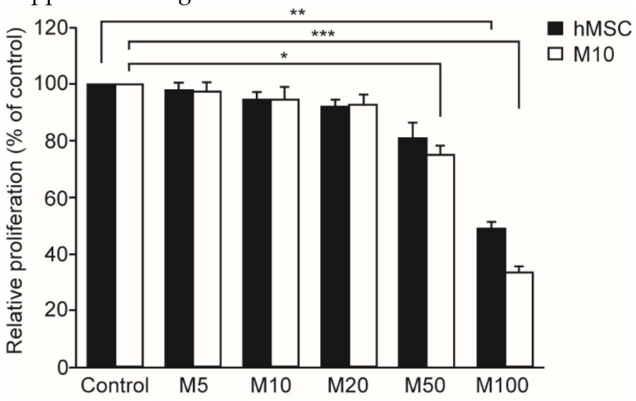

Supplemental Figure S1. High dose treatment of MDZ inhibits proliferation of normal cells. The hMSC (filled bars) and M10 cells (opened bars) were seeded overnight and then treated with MDZ (5, 10, 20, 50, 100 μM, denoted as M5, M10, M20, M50, M100, respectively) for 48 hours and the proliferation of cells was examined using MTT assay. The untreated cells were used as control. Results of three independent experiments are expressed as means ± standard deviation. *: P<0.05; **: P<0.01; ***: P<0.001.

Supplemental Figure S2

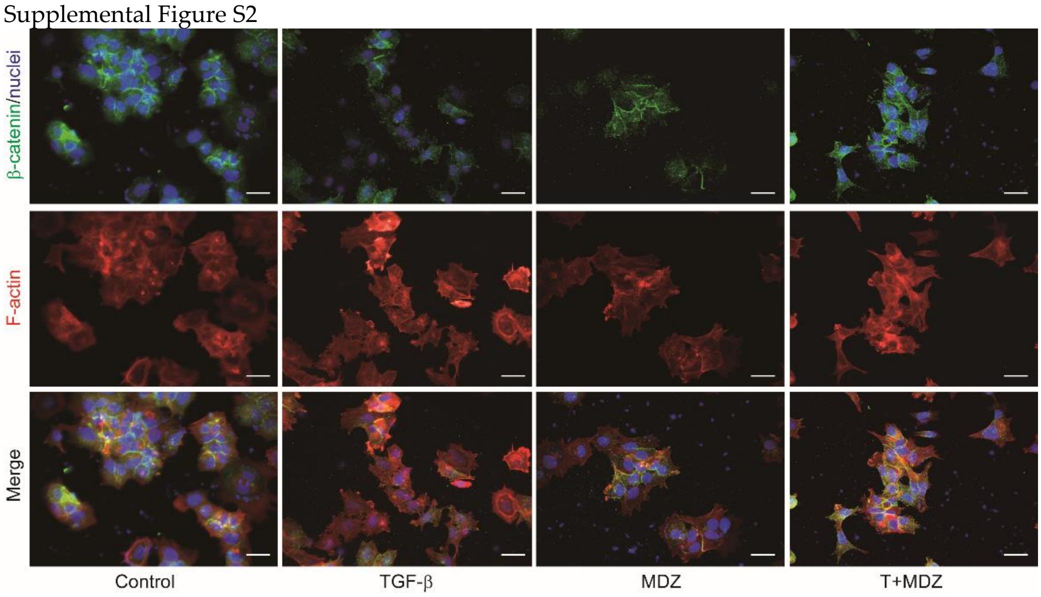

Supplemental Figure S2. Midazolam inhibits TGF-β-induced epithelial-mesenchymal transition in MCF-7 cells. Representative images of MCF-7 cells were grown on 20 μg/ml fibronectin-coated 22x22 mm coverslips and treated without (Control) or with TGF-β1 (10 ng/ml) (TGF-β) in the absence or presence of MDZ (20 μM) (denoted as MDZ and T+MDZ, respectively) for 24 hours. The localization of β-catenin and F-actin were determined by immunofluorescence. The β-catenin was stained with Alexa-488 (green) and F-actin was stain*ed* with phalloidin-TRITC (red). Nuclei were stained with DAPI (blue). Scale bar: 20 μm.
